# A Triple‐Layered Composite Scaffold of Silk Fibroin and Decellularized Amniotic Membrane for Bladder Tissue Engineering

**DOI:** 10.1002/mabi.202500157

**Published:** 2025-08-14

**Authors:** Melina Mamdoohi, Mehdi Shafieian, Zahra Hassannejad

**Affiliations:** ^1^ Department of Biomedical Engineering Amirkabir University of Technology (Tehran Polytechnic) Tehran Iran; ^2^ Pediatric Urology and Regenerative Medicine Research Center Gene, Cell and Tissue Research Institute Tehran University of Medical Sciences Tehran Iran; ^3^ Biodiscovery Institute University of Nottingham Nottingham UK; ^4^ Department of Chemical and Environmental Engineering University of Nottingham Nottingham UK

**Keywords:** adipose tissue‐derived stem cells, amniotic membrane, bladder regeneration, tissue engineering, SF

## Abstract

Augmentation cystoplasty has different side effects in urinary bladder reconstruction. Accordingly, it is necessary to develop substitutes using natural and synthetic biomaterials to address current problems. This study evaluates the potential of a triple‐layered composite scaffold for bladder regeneration. The triple‐layered scaffold consists of a silk fibroin (SF) film blended with polyethylene oxide (PEO), a decellularized human amniotic membrane (DHAM), and a lyophilized SF sponge, which is seeded with adipose tissue‐derived stem cells (ADSCs) encapsulated in collagen hydrogel. The mechanical properties of the triple‐layered scaffolds closely resemble those of human bladder tissue. The cell survival, proliferation, and viability of the different layers of the scaffold are assessed. The results show that DHAM and silk sponge at a concentration of 4% wt v^−1^ achieve a high level of biocompatibility. To study potential stone formation, scaffolds either with DHAM or without DHAM are exposed to human urine. Field emission scanning electron microscopy (FESEM) and X‐ray diffraction analyses indicate that the scaffolds with DHAM do not exhibit any signs of erosion or the creation of crystalline particles after 7 days. In conclusion, the data presented in this study highlight a new triple‐layered scaffold for the purpose of bladder tissue engineering.

## Graphical abstract

This study develops a triple‐layered scaffold for bladder regeneration using a SF/PEO film, decellularized human amniotic membrane, and a SF sponge seeded with ADSCs encapsulated in collagen hydrogel. The scaffold demonstrates excellent biocompatibility, mechanical properties resembling bladder tissue, and resistance to urinary stone formation, making it a promising candidate for bladder tissue engineering applications.

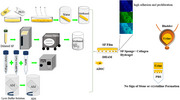

## 1 Introduction

The urinary bladder is susceptible to various congenital and acquired defects, including neurogenic bladder, bladder exstrophy, poor compliance, overactive bladder, cancer, trauma, infection, and inflammatory conditions [1, 2]. At present, the use of autologous gastrointestinal segments is the gold standard for bladder reconstruction and is associated with significant complications such as chronic urinary tract infection, metabolic disturbances, urolithiasis, stone formation, increased mucus production, and secondary malignancies.[3, 4] Hitherto, tissue engineering approaches have been investigated in detail, focusing on the selection of scaffold materials, their modification, and the choice of cell type for seeding on scaffolds. Additionally, studies have investigated the regeneration of bladder tissue using a variety of synthetic polymer scaffolds, such as polylactic/polyglycolic acid, polyethylene, and polyvinyl, as well as natural polymers like silk, chitosan, gelatin, alginate, and natural scaffolds derived from native tissues, for instance decellularized bladder acellular matrix (BAM), small intestinal submucosa, and amniotic membrane (AM). These studies have explored the use of these scaffolds alone or in combination with various types of tissue‐specific cells and stem cells in different animal models. According to the obtained data, these scaffolds are limited because of urinary tract infections, urolithiasis, graft contracture, and rejection. However, the development of engineered bladder tissues has the potential to bring significant advancements in the treatment of bladder disorders [5, 6, 7, 8, 9]. In conclusion, scaffolds must possess properties suitable for constructing the urinary barrier, including biocompatibility, biodegradability, and mechanical properties closely resembling those of native tissue. Moreover, scaffolds must be easily surgically manipulated to choose the optimal scaffold in order to minimize leaks and reduce the occurrence of local inflammatory responses. They must also possess sufficient mechanical resistance to withstand the forces necessary for bladder contraction [2, 10].

Silk fibroin‐based biomaterials have several advantageous characteristics, including high structural strength, elasticity, tunable biodegradability, and appropriate inflammatory responses, making them effective for the preservation of bladder function [11, 12, 13, 14]. Notably, recent studies have demonstrated the effective use of silk scaffolds in supporting bladder tissue repair in rat and pig models of augmentation cystoplasty [15, 16, 17, 18]. Over recent years, AMs have been employed as grafts for bladder tissue regeneration and have shown successful results in bladder augmentation [19, 20, 21, 22, 23, 24]. The AM possesses several advantageous biological properties, including anti‐inflammatory, antimicrobial effects, and anti‐fibrotic activates, and it is easily available for tissues repair [25, 26, 27, 28, 29].

Additionally, among stem cells, adipose tissue is rich in mesenchymal stem cells (MSCs), commonly referred to as adipose‐derived stem cells (ADSCs) which have the ability to accelerate the process of tissue repair [30, 31, 32]. In addition to the advantages previously mentioned, the culture of ADSCs in collagen hydrogel has been demonstrated to be effective in repairing and regenerating tissue [33]. Various studies have reported that ADSCs have the potential to differentiate into multiple mature cell types, such as urothelium, osteocytes, and lipocytes. Furthermore, it has been observed that ADSCs possess the ability to secrete various growth factors, including angiopoietin‐1, vascular endothelial growth factor, nerve growth factor, brain‐derived neurotropic factor, and glial cell‐derived neurotrophic factor, which promote in vitro angiogenesis and the growth of nerve axons [30, 34]. As a result, ADSCs have the potential to be extensively used in tissue engineering for bladder regeneration [31, 32, 35, 36, 37, 38, 39].

In this study, the efficacy of a triple‐layer scaffold comprising a SF film, a SF sponge layer, and a decellularized human amniotic membrane (DHAM) was evaluated. ADSCs encapsulated in collagen hydrogel were also entrapped within the scaffold to promote bladder tissue regeneration [33, 40]. In addition, both the three and two‐layer scaffolds were challenged with human urine to identify potential stone formation. Although numerous previous studies have investigated various combinations of SF scaffolds, either with or without the presence of stem cells, they have been constrained by issues involving bladder stone formation, reduced elasticity and stiffness of the regenerated tissue, inflammatory reactions, diminished compliance, and suboptimal degradability of the scaffold [15, 16, 17, 18]. Considering these limitations, the current study proposes a multi‐layered scaffold consisting of three distinct biomaterials and stem cells. Certain studies have explored the combination of silk films with PEO, demonstrating favorable morphological and mechanical characteristics suitable for tissue engineering applications [41, 42, 43, 44, 45]. However, specific studies have shown that lyophilized scaffolds are effective materials for both soft and hard tissue engineering projects [46, 47]. Moreover, AM possesses a suitable substrate that is characteristic of bladder tissue. We use a layer of DHAM to protect against direct contact of urine to silk scaffolds and prevent inflammatory reactions and risk of stone formation. Human amniotic membrane (HAM) was decellularized to enhance its biological properties while simultaneously reducing immunogenicity through the removal of cellular components. This decellularization allowed for improved biological characteristics and cell survival by exposing extracellular proteins. As this layer will be directly exposed to urine, it was essential to decellularize the HAM to minimize the risk of inflammatory reactions and stone formation. In addition, studies have demonstrated that ADSCs have consistently produced reliable results, and collagen is capable of supporting their survival [30, 34].

Previous studies have employed bi‐layered scaffolds composed of SF films and SF sponge layers for bladder augmentation in rats and pigs [15, 16, 17, 18]. These studies reported de novo tissue formation of smooth muscle layers, vascularization in all regenerated tissues, and innervation processes, alongside the formation of multi‐layered urothelia with prominent UP3A and pan‐CK protein expression. However, such constructs faced certain challenges, such as high stiffness of the scaffolds, increased incidence of stone formation, slow degradation rates of SF films, and a potentially inflammatory response. The most significant challenge faced by these previous studies was related to the non‐optimized film degradation and inflammatory response caused by the SF scaffolds. Both these factors were observed to be the most significant obstacles, leading to the formation of stone and inflammatory responses [15, 16, 17, 18]. In order to improve the scaffold, we aimed to optimize the degradation rate of the SF film and reduce the inflammatory response while also increasing the flexibility of the scaffold, allowing for ease of use during implantation.

To address these challenges, we added a DHAM layer to avoid inflammation and promote an even more biocompatible approach. Moreover, by blending PEO with the SF film, we were able to optimize degradation rates. These modifications helped to create a more suitable scaffold for bladder tissue regeneration.

We hypothesized that bladder regeneration could be effectively achieved through the utilization of the triple‐layered scaffold, which incorporates a combination of SF film and sponge, DHAM, and ADSCs encapsulated in collagen hydrogel. The objective of this strategy is to enhance biodegradation control, improve mechanical properties, biocompatibility, eliminate inflammatory reactions, prevent stone formation, and protect cell proliferation (Supporting Information).

## 2 Materials and Methods

### 2.1 Experimental Materials

Sodium carbonate (Na_2_CO_3_), methanol (CH_3_OH), sodium hydroxide (NaOH), TRIS ((HOCH_2_)_3_CNH_2_), sodium dodecyl sulfate (SDS), EDTA (C_10_H_16_N_2_O_8_), Hydrochloric acid (HCl), Sodium chloride (NaCl), HNa_2_O_4_P_12_H_2_O, Acetone (C_3_H_6_O), Isopropanol (C_3_H_8_O), Acetic acid (CH_3_COOH), and 4’, 6‐diamidino‐2‐phenylindole (DAPI) were purchased from Merck Millipore, Germany. Lithium bromide (LiBr), poly (ethylene oxide) (PEO, M_W_ = 900 000 g mol^−1^), and (3‐(4, 5‐dimethylthiazol‐2‐yl)‐2, 5‐diphenyl tetrazolium bromide) (MTT) were purchased from Sigma–Aldrich, USA. Bombyx mori cocoons were provided by the Iran Silk Research Center. The cell culture medium (DMEM/F12), fetal bovine serum (FBS), trypsin, penicillin, streptomycin, and amphotericin B were purchased from Gibco. All the materials were used as received and used as such without further purification.

### 2.2 Preparation of Silk Fibroin (SF) Solution

SF was extracted from Bombyx mori cocoons by degumming pieces of cocoons in sodium carbonate solution (0.02 м) for 30 min to eliminate sericin. The purified SF was dissolved in aqueous lithium bromide solution (9.3 м) for 4 h at 60°C. The solution was dialyzed against deionized water using a dialysis cellulose membrane (12–14 kDa MW pore size, Sigma–Aldrich, USA) to obtain 8% wt v^−1^ purified SF solution. The SF solution was stored at 4°C [48].

### 2.3 Fabrication of SF/PEO Films

Blend films were prepared by mixing various concentrations of PEO (i.e., 0.1, 0.163, 0.4, 0.6 wt v^−1^ %) with 8 wt v^−1^ % SF solution in a 1:1 volume ratio. The resulting mixture was dispensed into wells of standard 24‐well cell culture plates and allowed to dry under ambient conditions. To improve the stability of the films, they were immersed in an aqueous solution of methanol (90% v v^−1^). Finally, the PEO was leached out from films by immersing them in a water bath at room temperature for 24 h.

### 2.4 Fabrication of SF Sponges

The SF solution was diluted to 4% and 6% wt v^−1^ and dispensed into the wells of standard 24‐well cell culture plates to create scaffolds. The diluted solutions were frozen overnight at −20°C and lyophilized for 24 h. The dried scaffolds were removed from the plate and treated with methanol to render them insoluble in aqueous environments.

### 2.5 Preparation of Collagen Hydrogel

Collagen was extracted from rat tail tendon using a previously published protocol [49]. Briefly, collagen fibers were soaked in acetone and 70% isopropanol before adding to acetic acid and shaken for 48 h. The extracted collagen was poured into the plastic plate molds and lyophilized. The resulting collagen was dissolved in 0.1 м acetic acid to a concentration of 6 mg mL^−1^.

In order to prepare the hydrogel, solutions of 1.3 м NaCl, 0.2 м HNa_2_O_4_P_12_H_2_O, DMEM/F12 (10x), and 0.3 м NaOH were added to the collagen solution. Typically, 100 µL of DMEM/F12 and 100 µL of NaCl + HNa_2_O_4_P_12_H_2_O solutions were added to 1 mL collagen solution at 4°C, and the pH of the solution was adjusted by adding 300 µL of 0.3 м NaOH The gelation process was initiated by increasing the temperature to 37°C and incubating the mixture at this temperature for 40 min.

### 2.6 Decellularization of Human Amniotic Membrane (HAM)

Decellularized human amniotic membrane (DHAM) was obtained using a method described in our previously published study [50]. Briefly, the HAM was preserved in phosphate‐buffered saline (PBS) solution containing gentamycin for 24 h. The HAM was then soaked in lysis buffer solution (containing TRIS and EDTA) for 1 h. Next, the HAMs were decellularized by placing them in 0.1% sodium dodecyl sulfate solution on a shaker plate for 48 h at room temperature. Finally, the membranes were flushed three times with PBS to remove residual detergent [50].

### 2.7 Fabrication of Triple‐Layered Scaffolds

**FIGURE 1:**
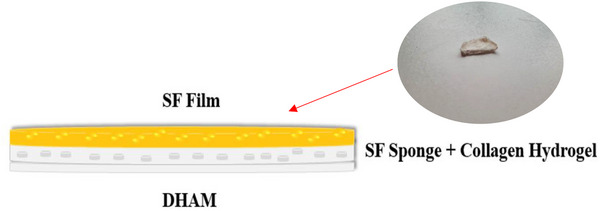
The image shows a triple‐layered scaffold, including the SF film, SF sponge + collagen hydrogel, and DHAM.

SF solution was used as a glue to attach SF film to SF sponge. The adhesion of DHAM to this triple‐layered scaffold was achieved using a collagen solution that was applied to the other surface of the SF sponge. The triple‐layered scaffold was then incubated at 37°C for collagen hydrogel formation and further attachment of layers (Figure 1).

### 2.8 Isolation and Culture of Adipose‐Derived Stem Cells (ADSCs)

All procedures were approved by the Animal Ethics Guidelines of Tehran University of Medical Sciences. Adipose‐derived stem cells were extracted from transgenic rats that expressed green fluorescent protein (GFP). ADSCs were isolated from the subcutaneous and the inguinal adipose tissues of adult Wistar rats under sterile conditions. The tissues were washed thrice with sterile PBS and minced into small pieces. Tissue explants were cultured in DMEM/F_12_ supplemented with 10% FBS and 2% penicillin/streptomycin and incubated at 37°C and 5% CO_2_ until the outgrowing cell confluency reached 80%.

ADSCs were characterized at the 3th passage using flow cytometry (BD FACSCalibur, BD Biosciences, USA) for MSC markers (CD105 and CD90) and hematopoietic marker (CD34). Experiments were performed using cells between passages 3 and 5 [51].

### 2.9 Cell Seeding

We shaped each of the scaffold groups to fit the dimensions of a 24‐well plate and then carefully seeded the cells over the surface of each group. ADSCs were seeded on different scaffold groups including SF/ 0.16% PEO ، SF‐S 6%, SF‐S 4%, and SF‐S 4% with collagen hydrogel and DHAM, at a cell density of 1 × 10^5^ cells/scaffold in 24‐well cell culture plates (Table 1).

**TABLE 1:** The codes were assigned to different scaffold groups in various concentrations of PEO and SF solutions for the prepared SF films and sponges.

Code	SF/0.10% PEO	SF/0.16% PEO	SF/0.40% PEO	SF/0.60% PEO	SF–S 4%	SF–S 6%
Description	Silk fibroin 8% wt vol^−1^ +PEO solution 0.10% wt vol^−1^ (Film)	Silk fibroin 8% wt vol^−1^ +PEO solution 0.16% wt vol^−1^ (Film)	Silk fibroin 8% wt vol^−1^ +PEO solution 0.40% wt vol^−1^ (Film)	Silk fibroin 8% wt vol^−1^ +PEO solution 0.60% wt vol^−1^ (Film)	Silk fibroin sponge 4% wt vol^−1^	Silk fibroin sponge 6% wt vol^−1^

To prepare SF‐S with collagen hydrogel group, the hydrogel was formed by adding collagen solution to the sponge and incubating it at 37°C and 5% CO2 for 40 min. The ADSCs were then seeded onto the sponges and other scaffolds.

### 2.10 Fabrication of Chamber for Evaluation of Stone Formation

**FIGURE 2:**
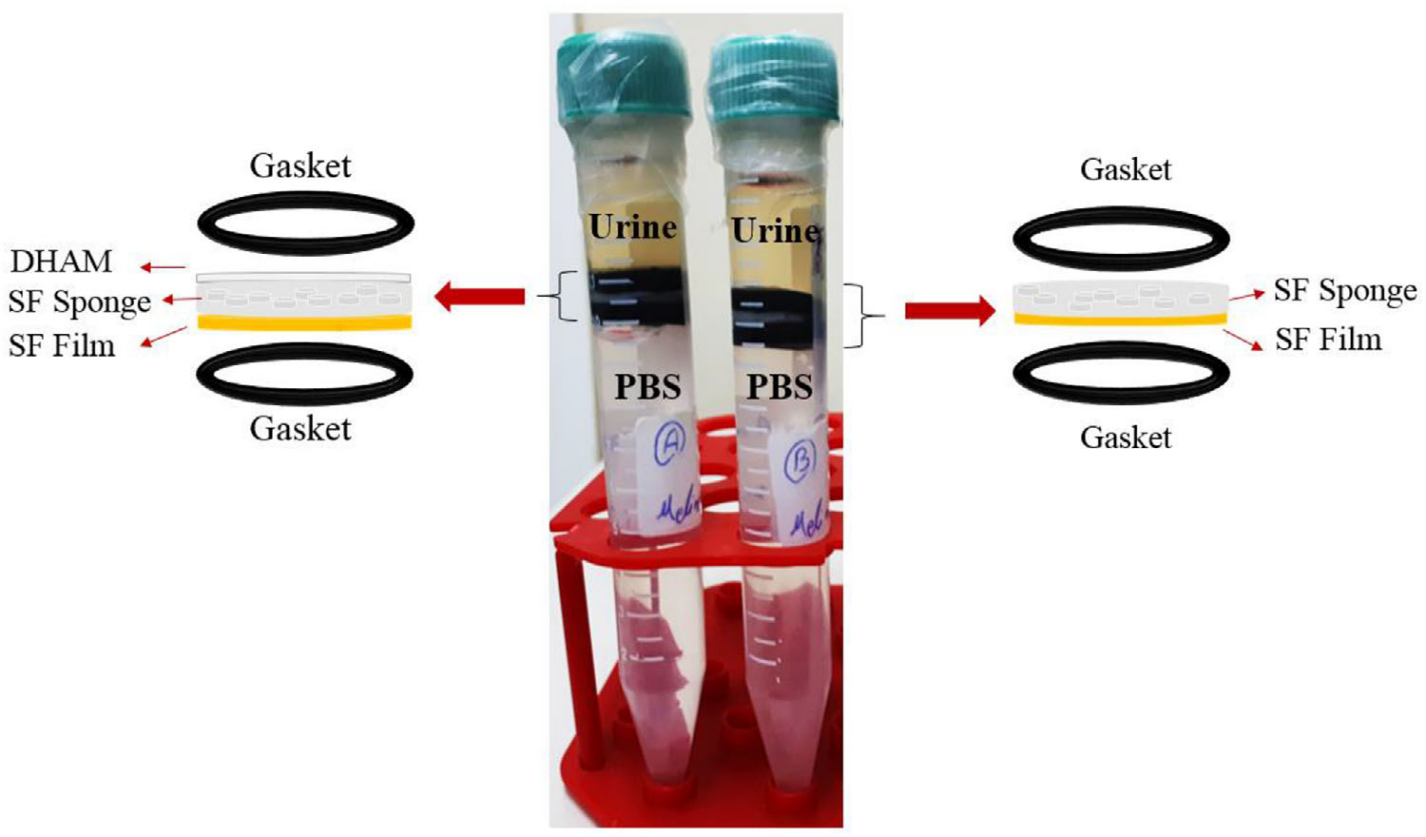
Fabricated chamber to evaluate bladder stone formation in the left falcon DHAM and right falcon SF‐S 4% exposed to human urine.

To investigate stone formation after scaffold implantation into the bladder, a chamber designed for observation was developed, as illustrated in Figure 2. Two groups of scaffolds, two layer and three layer, were inserted into Falcon 15 mL between the two gaskets (Table 2). Two groups of scaffolds were examined, with one side of the films in contact with PBS, and the other surfaces of the sponge or decellularized human amniotic membrane (DHAM) exposed to human urine. This setup was then placed in a warm water bath at a controlled temperature of 37°C for a duration of 7 days.

**TABLE 2:** Two separate scaffold groups were inserted into the chamber and exposed to human urine to simulate a bladder‐like environment.

Group	Two layer scaffold	Three layer scaffold
Description	Contain of SF/0.16% PEO and SF–S 4%	Contain of SF/0.16% PEO, SF–S 4%, and DHAM

For assessing the potential for stone formation in two‐ and three‐layer scaffolds, we measured the urine pH of the scaffolds after exposure to urine. We submerged both groups of two (sponge layer exposed to urine) and three (DHAM exposed to urine) layer scaffolds in human urine for 3 and 7 days. The pH of the urine was measured using a pH meter (Hanna HI2211, Italy) after each exposure period.

## 3 Characterization of Scaffolds

### 3.1 Attenuated Total Reflection‐Fourier Transform Infrared Spectroscopy (ATR‐FTIR)

Samples were dried and kept in a desiccator for at least 3 days to minimize the intervention of water peaks during FT‐IR analysis. FTIR spectra for verification of secondary structures in scaffolds and determination of the molecular conformation were recorded using an EQUNIOX 55 infrared spectrum analyzer via an attenuated total reflectance (ATR) accessory (Bruker, Germany). For each measurement, 64 scans with a resolution of 4 cm^−1^ were coded with a scanning step range of 650–4000 cm^−1^.

### 3.2 X‐Ray Diffraction Analysis

The Crystalline structure of the scaffolds was analyzed using the X‐Ray‐Diffractometer INEL (EQUINOX 3000, France). Ni‐filtered Cu‐Kα radiation was used as an X‐ray source under the conditions of 40 KV and 30 mA. Data were collected in the range of 2θ = 5°–80° at a speed of 2 min^−1^.

### 3.3 Field Emission Scanning Electron Microscopy (FE‐SEM)

The microstructure of the scaffolds was analyzed using FE‐FEM imaging technique. Scaffolds were sputter‐coated with a thin layer of gold prior to imaging. To assess the cross‐section of the triple‐layered scaffolds, the scaffolds were quenched in liquid nitrogen to obtain an interior cross‐section and then sputter‐coated with a thin layer of gold. FESEM images were recorded via a Field emission scanning electron microscope (ZEISS Sigma 300, Germany). Moreover, to observe the morphology of ADSCs seeded on the scaffolds after 72 h, the cells were fixed using 4% paraformaldehyde 4 and serially dehydrated using ethanol. FE‐SEM images were analyzed using ImageJ software. Pore size and porosity of scaffolds were calculated by ImageJ software. Surface features were counted from one representative image for each sample using the manual trace tool in ImageJ. Finally, using Excel 20 values were randomly selected and means and standard deviations (SD) were calculated.

### 3.4 Mechanical Properties

Uniaxial tensile tests were operated using the static mechanical testing machine (Instron 5566 UTM, US) and 100 N capacity load cell. Scaffolds were cut into rectangular strips with dimensions of 14 mm × 5 mm (height × width) and then were hydrated in PBS for at least 24 h at room temperature prior to testing. Three scaffolds were analyzed in each group.

A displacement control mode was used for testing, with a crosshead displacement rate of 5 mm/min, and the sample's initial gauge length was set to 10 mm. The initial elastic modulus (EM), ultimate tensile strength (UTS), and % elongation to failure were obtained from stress/strain plots. EM was calculated by using a least‐squares fitting between 0.02 N load and 5% strain past this initial load point. The UTS was defined as maximum stress during the test and the % elongation to failure was the last data point prior to failure.

### 3.5 Histological Analysis

To evaluate the decellularization process, the amniotic members, before and after decellularization, were stained with hematoxylin and eosin (H&E). Briefly, the tissues were washed with PBS and fixed in 4% paraformaldehyde at room temperature for 24 h. The samples were then dehydrated through a series of graded alcohols before being embedded in paraffin wax. Slices with a thickness of 5 µm were cut and stained with H&E.

### 3.6 MTT Assay

The effect of different scaffolds on cell viability was assessed using MTT assay in groups of scaffold including DHAM, SF‐S 4%+ Collagen, SF‐S 4%, SF‐S 6%, and SF/0.16% PEO. The ADSCs in tissue culture plates without any scaffold were set as a control. Cells were seeded in 96‐well plates at a density of 2 × 10^4^ cells/well and after 72 h, 80 µL of MTT solution (5 mg mL^−1^) was poured into the wells and incubated at 37°C for 4 h. Appeared formazan crystals from the cleavage of MTT were dissolved in 100 µL DMSO for 5 min with shaking, and finally absorbance at a wavelength of 570 nm was measured using a microplate reader (BioTek, Elx800, USA).

### 3.7 DAPI Staining

To investigate adherence and population of cells on the scaffolds, the cells’ nuclei were stained using DAPI (4’, 6‐diamidino‐2‐phenylindole). About 72 h after cell seeding, the scaffolds were fixed in 4% formalin, washed in PBS, and the nuclei stained with DAPI (1:5000) for 20 min. The images were captured using a fluorescence microscope, and three images were analyzed from each scaffold. Cells were counted in the DAPI images by ImageJ software. For each sample, five images were selected, then cell counting, mean and SD were obtained by Excel. Then data log‐normalized to produce a normal distribution of data by one‐way ANOVA.

### 3.8 Statistical Analyses

Data are expressed as mean ± SD. Statistically significant differences between data were evaluated via one‐way analysis of variance (ANOVA) with Graph Pad Prism software version 9.4, where p < 0.05 was considered to be significant.

### 3.9 Ethical Approval

We conducted all animal experiments in compliance with the ethical guidelines established by the Tehran University of Medical Sciences (TUMS), and the protocols for animal care were approved by the TUMS Ethics Committee (IR.TUMS.MEDICINE.REC.1399.668). We also adhered to the standards set out in the ARRIVE guidelines for reporting the use of experimental animals. Human urine and HAM were collected after obtaining informed consent from the donors or their legal guardians. The procurement of HAM and human urine strictly adhered to the regulations and protocols stipulated by the Ethics Committee of the TUMS.

## 4 Result

### 4.1 ATR‐FTIR Analysis

**FIGURE 3:**
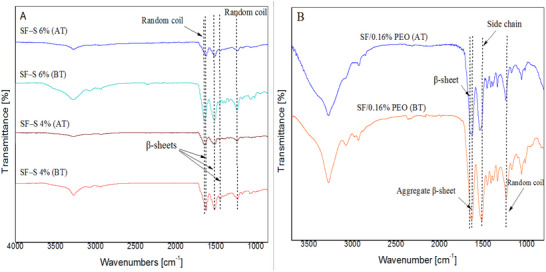
ATR‐FTIR spectra of (A) SF‐S 4% and SF‐S 6% and (B) SF/0.16% PEO film before and after methanol treatment. The peaks at 1636 and 1623 cm^−1^ (amide I) and 1445 cm^−1^ (amide II) are characteristic of secondary structure of silk II. Moreover, the peaks at 1538 cm^−1^ in the prepared films are indicative of silk I structure.

Silk fibroin has two main structures: silk I, which contains random coils and amorphous regions, and silk II, which contains antiparallel β‐sheet structures. Generally, silk I is soluble in water and can be transformed into a water‐insoluble silk II structure through chemical methods such as methanol treatment. In the literature, amide I *(‐CO‐ str*etching vibrations), II (secondary *‐NH‐* bending), and III *(‐CN‐*stretching and *N─H* bending) were observed at 1600–1700, 1450–1600, and 1200–1300 cm^−1^, respectively [52, 53, 54, 55]. As shown in Figure 3, the SF scaffolds had similar spectra for amide I, with peaks at 1623–1636 cm^−1^, amide II at 1509–1538 cm^−1^, and around 1230 cm^−1^ and 1444 cm^−1^ for amide III, respectively. In Figure 3, the peaks corresponding to the β‐sheet conformation appeared at 1636 and 1445 cm^−1^ in SF–S 4%, at three positions 1636, 1623, and 1446 cm^−1^ in sponge SF–S 6%, and at two positions 1636 and 1623 cm^−1^ in the SF/0.16% PEO film after methanol treatment.

Moreover, Figure 3 was shown that in SF–S 4% after treatment, the peaks at 1619 and 1538 cm^−1^ shifted to 1636 and 1509 cm^−1^, respectively, and in the SF/0.16% PEO, peaks at 1621 and 1509 cm^−1^ shifted to 1636 and 1519 cm^−1^, respectively, after methanol treatment. In addition, the 1638 and 1235 peaks in SF–S 6% before treatment were related to random coil structure. In other hand, random coils, side chains, and aggregated β‐sheets were observed in the SF/0.16% PEO before treatment. Consequently, two peaks at 1619 and 1621 cm^−1^ that were indicated by the aggregate β‐strand, turned to a strong β‐sheet (1636 cm^−1^) after scaffolds were treated. Additionally, the peaks at 3400 and 1030 cm^−1^ indicate *‐NH* bending and *C─O* bending, respectively [12, 55, 56]. In summary, these findings demonstrate the impact of methanol treatment on the transition of silk conformation from random coils and weaker β‐sheets to stronger β‐sheets. These shifts in the spectrum can be explained by the different hydrogen bonding and reorganization of silk structural chains.

**FIGURE 4:**
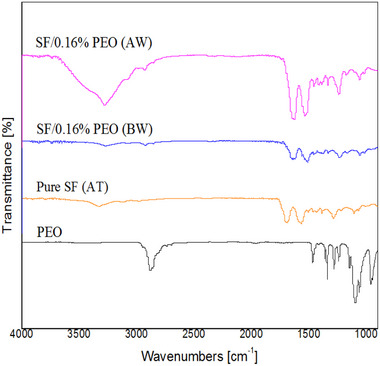
Comparison of ATR‐FTIR spectra of PEO powder, pure SF film, and SF/0.16% PEO films before (BW) and after washing (AW). PEO powder peaks were not observed in SF/0.16% PEO films after washing.

Figure 4 shows the ATR‐FTIR spectra of the PEO powder, pure SF film, and SF/0.16% PEO, before and after washing. As shown, there were no peaks related to PEO in SF/0.16% PEO after insertion into the water bath for 24 h.

### 4.2 X‐Ray Diffraction

**FIGURE 5:**
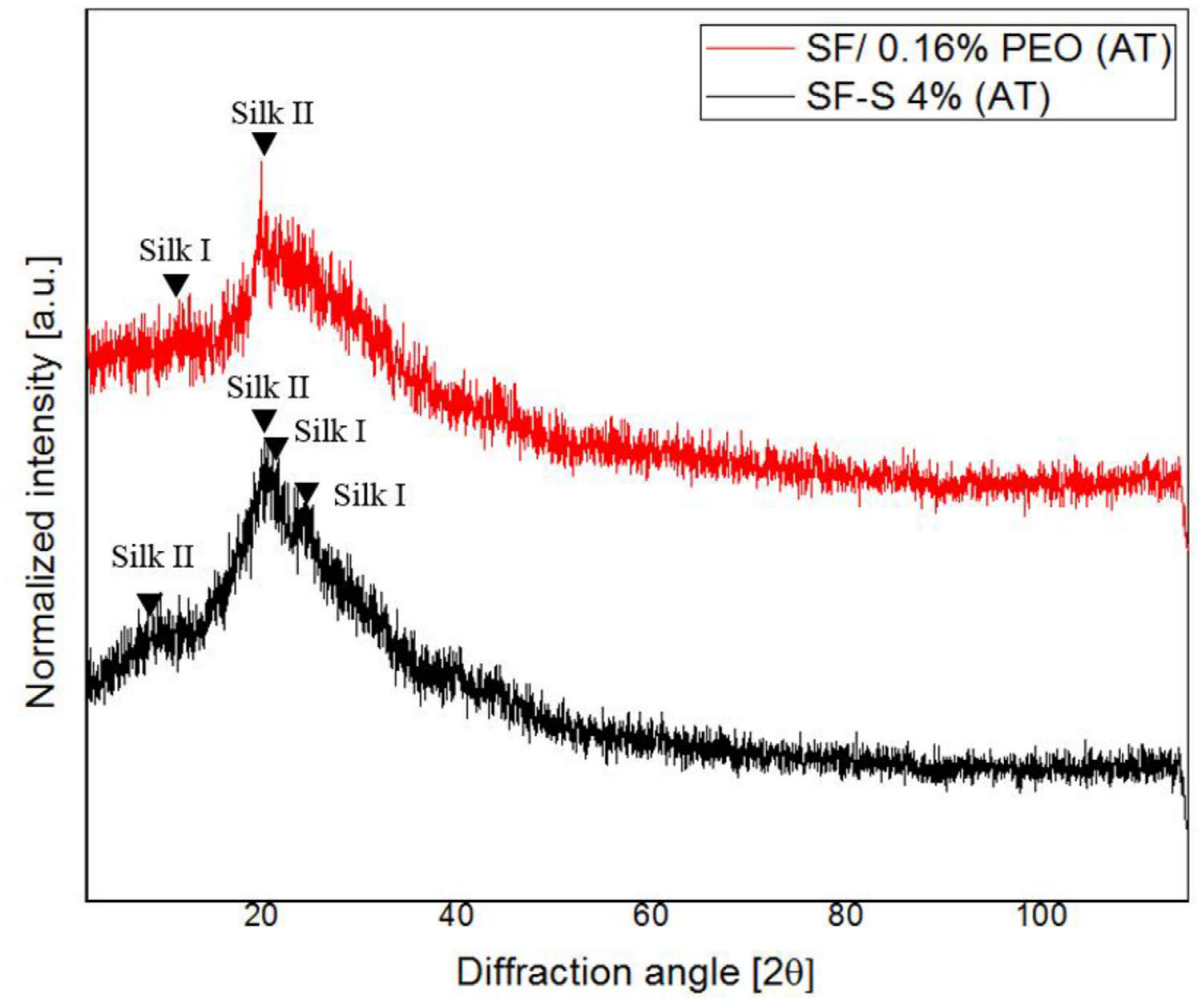
X‐ray diffraction (XRD) analysis of silk scaffolds. SF/0.16% PEO and SF–S 4% after methanol treatment (AT) exhibited structures of both silk I and silk II.

The XRD spectra in Figure 5 present the SF film and sponge after methanol treatment, which induced crystallization. The peaks detected at 10.94° and 20.37° (silk II and silk I, respectively) in SF–S 4% correspond to the diffraction peaks of silk II, while peaks at 20.05° belong to silk II, and at 11.91 and 24.69 are indicative of silk I. additionally, SF/0.16% PEO peaks at 19.91° and 12.57° determine silk II and silk I, respectively, in accordance with previous studies [12, 57]. The presence of diffraction peaks signifies a dominant silk II structure and verifies the presence of β‐sheet structures within the silk scaffolds [58]. Furthermore, it is notable that the silk scaffolds retained their silk I structure after methanol treatment, thereby increasing crystallinity. Therefore, methanol treatment can effectively adjust the degradation rate due to the dual presence of silk I and silk II structures within the scaffolds.

### 4.3 Investigation of Scaffold's Crystalline Structure Following Exposure Human Urine

**FIGURE 6:**
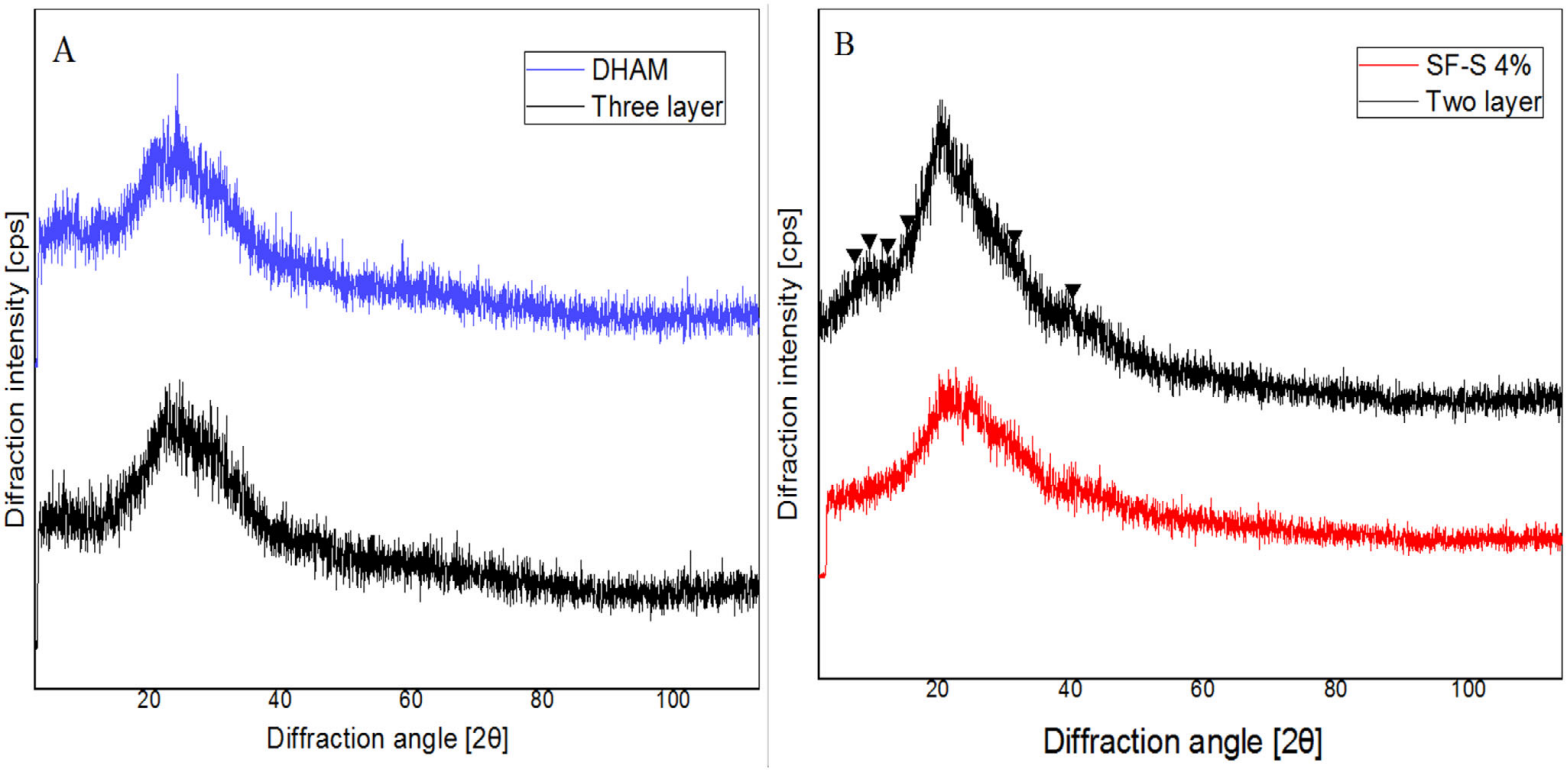
XRD analysis of the three (A) and two (B) layer scaffold groups. DHAM and three layer scaffolds had the same XRD pattern, but the XRD pattern of SF–S 4% was different from that of the two layer.

To assess the risk of stone formation in the bladder following scaffold implantation, XRD analysis was conducted on the two‐ and three‐layer scaffold groups that were exposed to human urine for a period of 7 days. As depicted in Figure 6, it is evident that the scaffold with two layers displays peaks in the 5°–15° and 30°–40° regions that were absent in the SF–S 4% after treatment as a control. In addition, the XRD patterns of DHAM in the three‐layer scaffold and DHAM as the control were the same.

### 4.4 Morphological Evaluation and FE‐SEM

**FIGURE 7:**
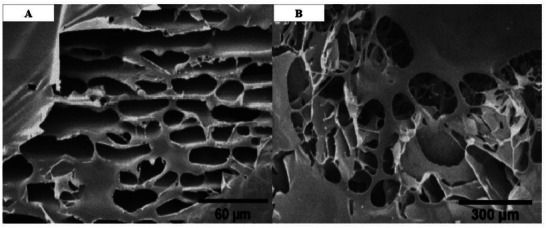
The Image shows the FE‐SEM images of the surfaces as porous sponges, with (A) representing the SF–S 6% and (B) representing the SF–S 4%. It is evident that the sponges with 6% SF solution concentration had a denser structure than sponges with 4% SF solution concentration.

The morphological study of the fabricated scaffold, consisting of three layers of DHAM, SF/0.16% PEO, and SF–S 4%, revealed a sponge‐like structure with sheet‐like lamellae and interconnected pores, as indicated in Figure 7. The micrograph demonstrates that the lyophilized silk sponges possessed these structural features. Notably, there was a twofold increase in the pore size in SF–S 4% compared to SF–S 6%, while the porosity of SF–S 4% reached 65% (Table 3).

**TABLE 3:** The porosity and pore size (diameter) of the sponges fabricated from 4% and 6% SF solutions were measured.

Scaffolds	S‐F 6%	S‐F 4%
Porosity (%)	47 ± 0.95	65 ± 0.74
*D* (µm)	106.10 ± 1.73	207.38 ± 1.66

**FIGURE 8:**
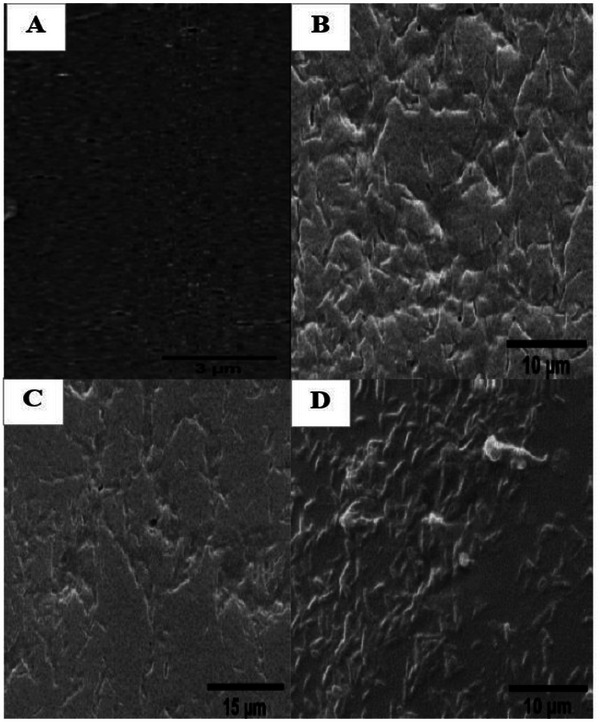
FE‐SEM images show the surface morphologies of the (A) SF/0.10% PEO, (B) SF/0.16% PEO, (C) SF/0.4% PEO, and (D) SF/0.6% PEO films.

Following the dissolution of PEO, the surface characteristics of the films were examined, with the resultant dent morphology attributed to PEO micro‐phase separation. However, the surface morphology of SF/0.10% PEO remained largely unaltered following PEO dissolution. Interestingly, the SF/0.16% PEO and SF/0.4% PEO films exhibited a pore‐like surface morphology. On the other hand, a globule‐like morphology was observed on the surface of the SF/0.6% PEO film, attributed to the elevated PEO concentration in the blend films during PEO phase separation. Concurrently, with the increase in PEO content, the pore size of the surface decreased. As depicted in Figure 8, the surface morphology of the films was influenced by varying PEO solution concentrations, resulting in diverse morphologies.

**FIGURE 9:**
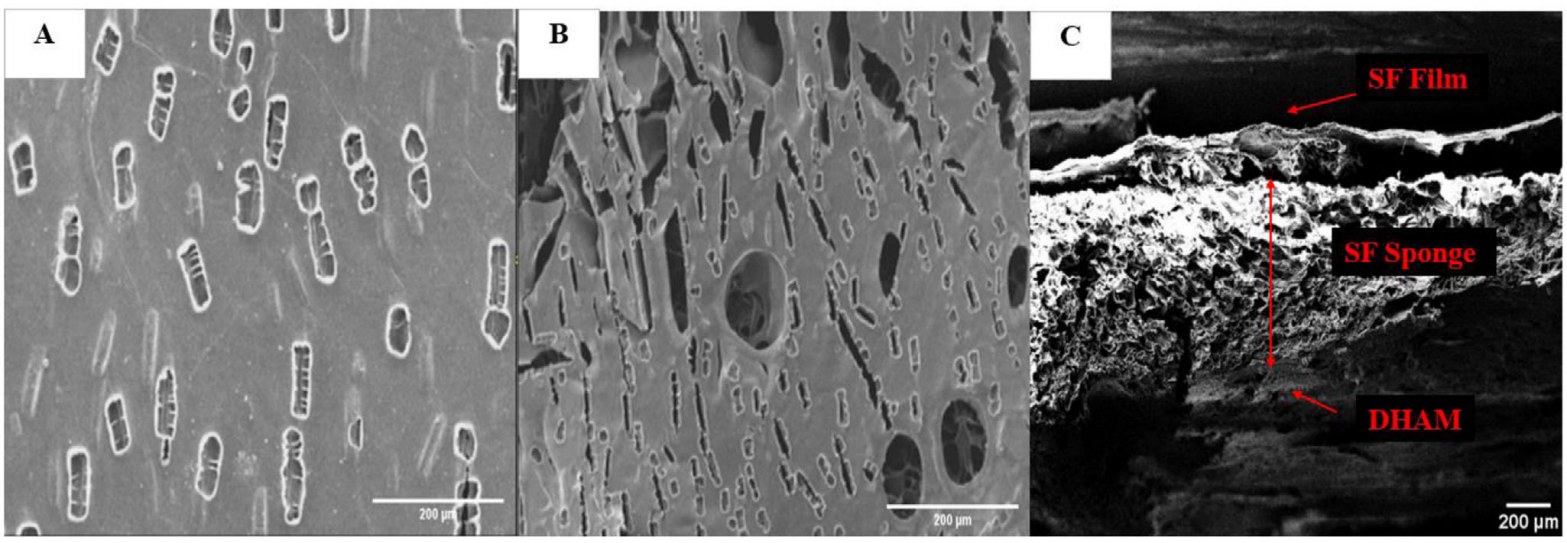
FE‐SEM cross‐sectional images of (A) SF/0.16% PEO, (B) SF–S 4%, and (C) the triple‐layer scaffold, where the latter demonstrates distinct layers comprising the silk film (outer layer), silk sponge (middle layer), and DHAM (inner layer).

Figure 9 displays cross‐sectional images of the SF/0.16% PEO film, SF–S 4%, and three‐layered scaffolds. The cross section of the film exhibited a depression‐like pore shape, whereas the cross section of the sponge revealed a porous structure. The cross‐sectional images of the triple‐layered scaffold confirmed the presence of the SF film, sponge, and DHAM layers from the top downward.

### 4.5 Structural Evaluation of Scaffolds Following Exposure Human Urine

**FIGURE 10:**
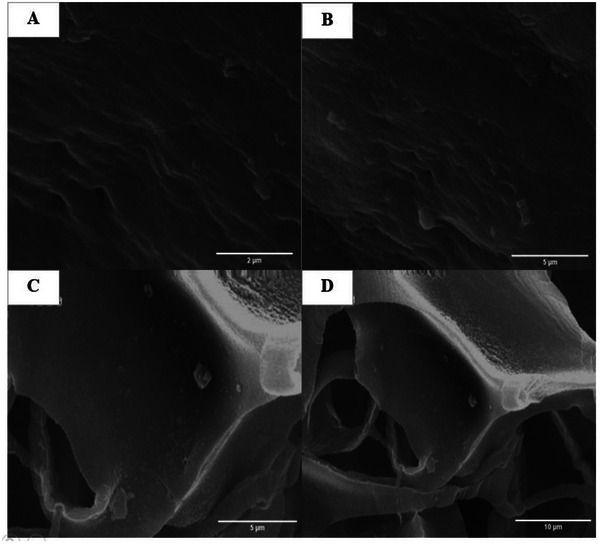
FESEM images of DHAM in three‐layer scaffold (A, B) and SF‐S 4% in two‐layer scaffold (C, D) following exposure to human urine for 7 days.

FE‐SEM images were captured to investigate the risk of stone formation following exposure to human urine for 7 days (Figure 10). Notably, solid phases and particles separated from the two‐layer scaffold group after 7 days of exposure, whereas no such solid phases or crystallite forms were detected in the three‐layer groups with DHAM in contact with human urine after the same duration.

### 4.6 Mechanical Properties

For comparison purposes, we referred to the articles that used similar settings including room temperature and rectangular strings.[59, 60] Additionally, we utilized the mechanical properties of DHAM, as obtained from a previous study to evaluate the DHAM under similar conditions in our experiment.[50] The findings indicated that SF/0.16% PEO and SF/0.1% PEO exhibited superior EM and UTS values compared to the other groups, as well as even human bladder tissue. This was attributed to the denser structure of the films. However, as the PEO concentration in the films increased, both UTS and EM values decreased, primarily due to the films' increased elasticity. The results showed that the blend films exhibited the highest ETF percentage when the PEO content was 0.16% and 0.6%. However, the film exhibited a compromised level of mechanical strength at a PEO concentration of 0.6%, falling short of the required performance for optimal mechanical stability. Consequently, a PEO concentration of 0.16% was deemed the more suitable option.

**FIGURE 11:**
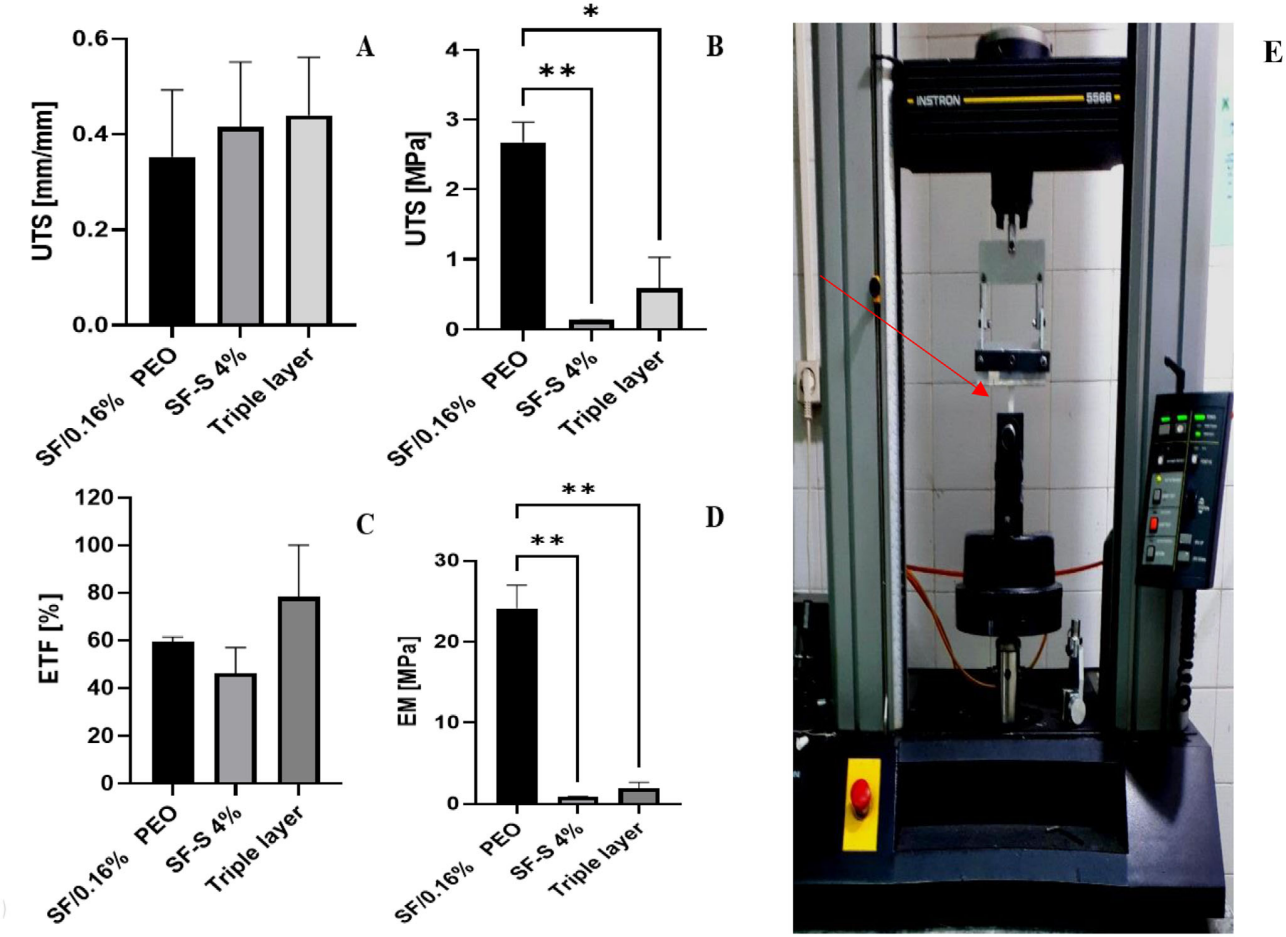
Mechanical properties of SF/0.16% PEO, SF‐S 4% and triple‐layered scaffold. Evaluation of (A) ultimate tensile strain (UTSm), (B) ultimate tensile strength (UTS), (C) elongation to failure (ETF), and (D) elastic modulus (EM), (D) the rectangular strip of the triple‐layered scaffold during the uniaxial tensile test. (n = 3, mean ± SD**),** *p* ≤ 0.05 (*)، ≤ 0.01 *p*(**). The Red arrow indicates the triple‐layered scaffold.

On the other hand, increasing the SF concentration in the sponges was associated with higher UTS values. The sponge with a 4% SF (SF‐S 4%) concentration exhibited a UTS and ultimate strain that was comparable to that of human bladder tissue, despite having a higher (3.6‐fold) EM. A past study revealed that AM materials had high‐UTS and EM values, though they exhibited lower ultimate strain. The ETF of the film and sponge was seen to be comparable to that of the human bladder. Prior to conducting the uniaxial test, we meticulously sutured the triple‐layered scaffolds together, ensuring no separation between the layers. Furthermore, the UTS, ultimate strain, and ETF of the triple‐layer scaffolds were found to be comparable to those of human bladder tissue, although the EM was 7.5‐fold greater due to the additional presence of the film and AM in the triple‐layered structure (Figure 11, Table 4). Additionally, the ETF of the triple‐layer scaffold was observed to surpass that of the film, sponge, and DHAM. This can be attributed to the necessity of an initial force for stretching the three layers together prior to the initiation of the force required for the layers to separate. After which, the force progressively increases until the layers experience rupture.

**TABLE 4:** Comparison of mechanical properties of different scaffold groups, the triple‐layered scaffold, human bladder, and rat bladder (n = 3, mean ± SD**)**.

Samples	SF/0.1% PEO	SF/0.16% PEO	SF/0.4% PEO	SF/0.6% PEO	SF‐S 4%	6 SF‐S 6%	DHAM	Triple layer	Human bladder	Rat bladder
UTS [MPa]	2.93 ± 0.1	2.68 ± 0.1	1.2 ± 0.15	0.51 ± 0.3	0.133 ± 0.02	0.23 ± 0.04	1.87 ± 0.16	0.316 ± 0.09	0.27 ± 0.14	0.72 ± 0.21
UTSm [mm/mm]	0.293 ± 0.03	0.354 ± 0.05	0.43 ± 0.02	0.62 ± 0.02	0.416 ± 0.03	0.34 ± 0.07	0.05	0.62 ± 0.08	0.69 ± 0.17	2.03 ± 0.44
EM [MPa]	33 ± 1.02	24.15 ± 1	22.34 ± 2.3	5.65 ± 0.7	0.906 ± 0.1	1.012 ± 0.08	45.95	1.87 ± 0.2	0.25 ± 0.18	0.76 ± 0.44
ETF [%]	40 ± 9	59.29 ± 11	51.82 ± 12	64 ± 8.2	46.12 ± 2, 87	37.88 ± 5.98	4.87 ± 0.66	78 ± 19	—	—
References	—	—	—	—	—	—	49	—	58, 59	58, 59

### 4.7 Hematoxylin and Eosin

**FIGURE 12:**
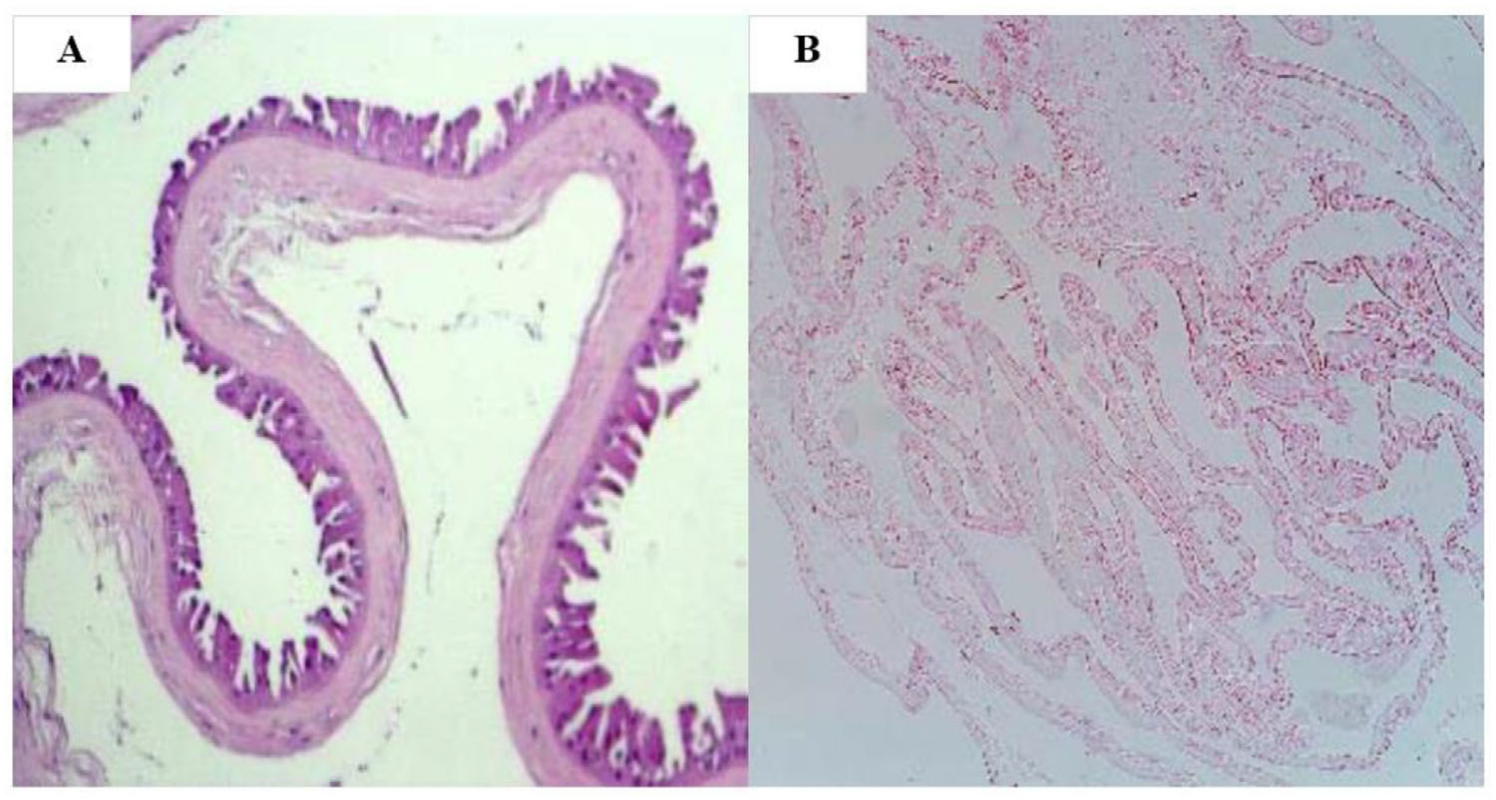
Microscopic images of H&E staining were taken of (A) fresh and (B) decellularized human amniotic membrane. The images clearly demonstrate that the cells were completely removed from the tissue after decellularization.

To confirm the effectiveness of the decellularization method in removing cellular components from the extracellular matrix of fresh AM, histological analysis was performed using hematoxylin and eosin (H&E). H&E staining revealed that the cells were completely removed from the human amniotic membrane and no cellular components were observed in theDHAM. (Figure 12). Additionally, based on a previous study, all necessary analyses have already been performed to validate the decellularization process [50].

### 4.8 Flow Cytometry

Flow cytometry analysis revealed that the cells were positive for MSC markers CD90 (86.3%) and CD105 (98.5%) and negative for hematopoietic marker CD34 (2.27%). According to previous reports, a positive expression above 85% and a negative expression below 5% indicate that the cells are MSCs and not hematopoietic cells.[51] Therefore, we confirmed that the extracted cells were indeed MSCs.

### 4.9 MTT Assay

**FIGURE 13:**
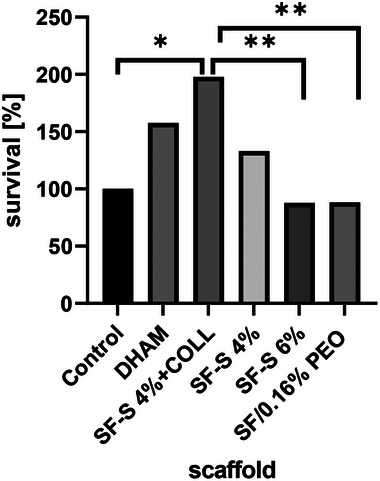
After 24 h of cell seeding, cell survival of the scaffolds was evaluated using the MTT assay. The results showed that the SF‐S 4% with collagen and AM exhibited the highest cell survival compared to the other groups.

The MTT assay exhibited that none of the scaffolds was cytotoxic to the cells. We conducted the MTT assay using standard conditions to accurately determine the number of cells that had grown within the scaffold groups, rather than on the plate itself. This allowed us to obtain reliable cell viability data and assess the efficacy of the scaffold in supporting cell growth. Figure 13 illustrates the biocompatibility of the scaffolds, which was evaluated by investigating cell survival after 24 h of ADSCs culture. All groups exhibited an increase in optical density after 24 h owing to the proper survival and proliferation of the cells on the scaffolds. SF‐S 4% with collagen hydrogel and DHAM had the highest OD in comparison to the other groups. Additionally, SF‐S 4% demonstrated increased cell survival compared to SF‐S 6%. It was concluded that SF‐S 4% with a more appropriate pore size for cells and enhanced survival rates was selected for the triple‐layered scaffold. Consequently, collagen was found to be effective in promoting cell survival and creating a favorable cell microenvironment for cell viability.

### 4.10 Cell Morphology, Adhesion and Proliferation on Layers of Scaffold

**FIGURE 14:**
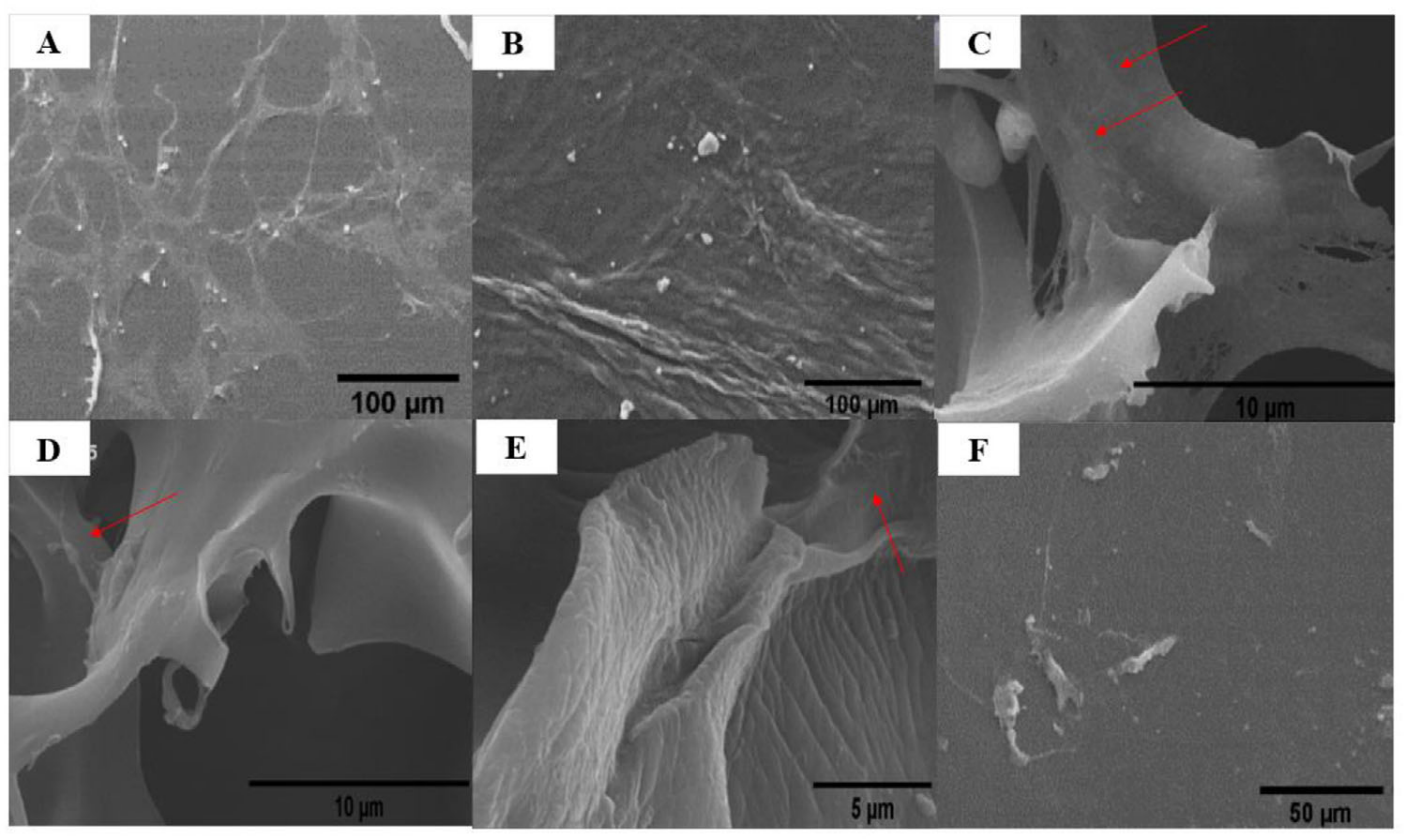
FESEM images of different groups of scaffolds after 72 h of ADSCs seeding. (A) Control, (B) DHAM, (C) SF‐S 4% +collagen, (D) SF‐S 4%, (E) SF‐S 6%, and (F) SF/0.16% PEO film. Arrows indicate ADSCs.

The morphology, adhesion, and proliferation of cells on the scaffold's layers were assessed after 72 h of ADSCs inoculation. Figure 14 shows that all groups of scaffolds exhibited cell adhesion and protected cell proliferation perfectly. Cell spreading and expansion were particularly notable on the silk scaffolds, particularly SF‐S 4% with collagen and DHAM. Firstly, it was confirmed that the concentration of the silk sponge combined with appropriate pore sizes provided an optimal environment for the cells. The SF/0.16% PEO film, characterized by its pore‐like structures and declining stiffness, demonstrated enhanced cell adhesion, making it a promising substrate for ADSCs. Following cellular seeding on the scaffolds for 72 h, cell morphology transformed into a spindle shape, indicating effective cell adhesion on all the scaffolds.

In general, FESEM imaging may not provide a fully reliable assessment of cell compatibility and viability, particularly after a short seeding period of 2 days. However, the primary objective of this analysis was to gain a general overview of cell morphology and cell adhesion to the various scaffold layers, rather than to conduct a comprehensive evaluation of cell viability or overall biocompatibility.

### 4.11 Immunofluorescent Assay

**FIGURE 15:**
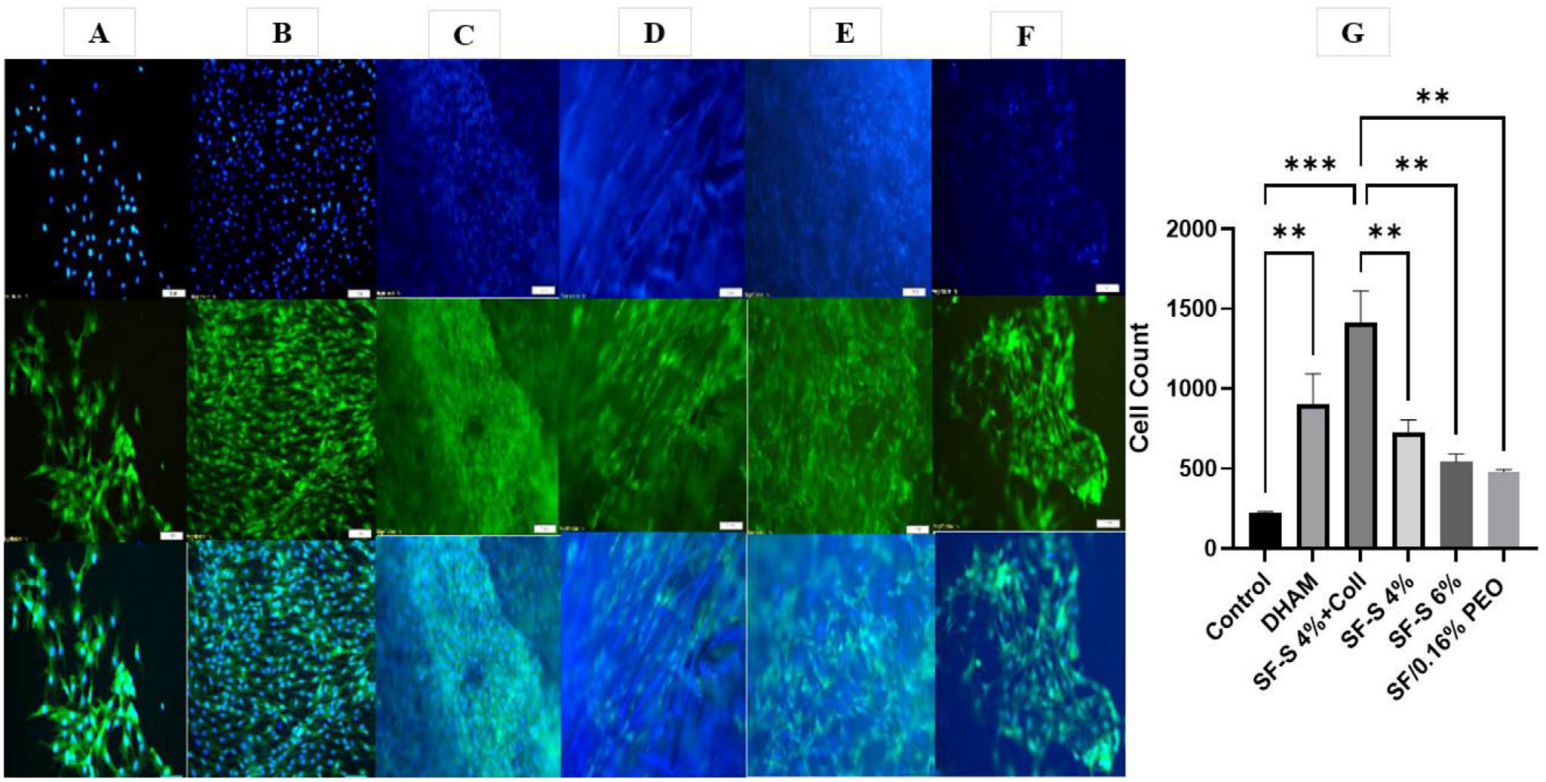
Fluorescent images and cell counts in all scaffold groups. DAPI, GFP, merged images (up to down) showing cell survival on scaffolds after 72 h of ADSCs culture. (A) Control, (B) DHAM, (C) SF‐S 4% +collagen, (D) SF‐S 4%, (E) SF‐S 6%, and (F) SF/0.16% PEO film (magnification of images at 1x. g). (G) Cell counts in different groups of scaffolds after 72 h of cell culture. The Number of cells was calculated by counting cells in five different fields in all scaffolds. Each value represents a mean ± SD. Significance was set at *p* < 0.05 (*) and *p* < 0.01 (**) with respect to control. Please not that due to the high fluorescent background in the blue channel and the porous nature of the sponge layers, image clarity was compromised.

Figure 15 demonstrates GFP‐positive ADSCs seeded on DHAM, SF‐S 4% +collagen, SF‐S 4%, SF‐S 6%, SF/0.16% PEO film, and tissue culture plate. It was observed that the cells demonstrated significantly higher adhesion and proliferation on SF‐S 4%+collagen due to the ideal porosity of the sponge combined with collagen hydrogel, which offered protection to the cells. DHAM also displayed exceptional biocompatibility and functioned as a native substrate for cellular interaction. In conclusion, all groups of scaffolds were found to be suitable for ADSC proliferation. However, SF‐S 4% with collagen hydrogel and DHAM exhibited the highest number of live cells, suggesting that the application of collagen hydrogel on the SF‐S 4% surface effectively enhanced cell survival and protection.

In Figure 15, due to the high fluorescent background in the blue channel, image clarity was somewhat compromised. Additionally, the porous structure of the sponge layers made it challenging to capture clear and detailed images. To address this, we applied a merging technique to enhance image clarity, allowing for improved visualization of the sponge structure and composition.

### 4.12 pH Measurement

**FIGURE 16:**
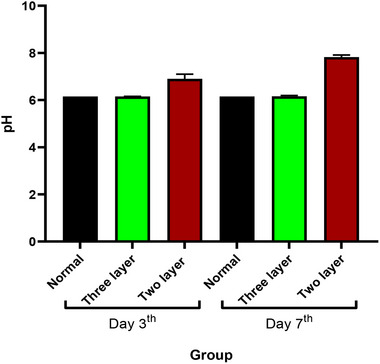
Evaluation of pH in three‐ and two‐layer scaffolds groups flowing expose to human urine after 3 and 7 days.

The pH was measured for human urine in two groups after 3 and 7 days. The pH of human urine was found to be 6.15, as illustrated in Figure 16. The pH of human urine in the two layer scaffold group was 6.92 after 3 days, while the three layer groups had a pH of 6.15. The pH of human urine in both groups was measured after 7 days; consequently, the two layer was observed with a pH of 7.83, and the three layer had a pH of 6.16.

## 5 Discussion

In patients with urinary bladder disease, the replacement of damaged tissue is essential. However, current surgical methods face significant challenges, including chronic infections, stone formation, and involuntary voiding of urine. Tissue engineering offers a promising alternative, and researchers are developing scaffolds to address both short‐term and long‐term complications associated with surgery [5, 6, 7, 8, 9].

Previous studies have explored various scaffolds, including polymer‐based scaffolds and acellular matrices, but these approaches have encountered limitations such as stone formation, inflammation, and suboptimal degradation times. To date, five primary approaches for bladder tissue engineering have been investigated: natural polymers (e.g., silk), synthetic polymers (e.g., poly‐glycolic acid (PGA)), naturally derived acellular matrices (e.g., BAM), cell‐based engineering, and composite scaffolds [5, 6, 7, 8, 9].

We hypothesized that a triple‐layered scaffold could serve as a suitable substitute for bladder tissue engineering. In this design, the film layer functions as a seal, the silk sponge supports cell growth, and the DHAM prevents direct contact with urine. This structure could mitigate stone formation while providing a platform for tissue regeneration. We conducted thorough testing on the produced scaffold, with plans to perform animal experiments to assess usability and effectiveness in a more realistic setting. The bladder is a complex organ consisting of multiple layers, making a triple‐layered scaffold potentially more accurate and acceptable for bladder regeneration.

### 5.1 Silk Scaffolds in Bladder Tissue Engineering

Various silk scaffolds, including dense and porous structures, have been applied in bladder tissue engineering [61, 62, 63, 64]. In the past decades, silk has been discussed broadly due to its biocompatibility, superior mechanical performance, biodegradation, and ease of processing. However, silk scaffolds have exhibited some drawbacks, such as slow degradation rate, unoptimized mechanical properties, inflammation reactions, and stone formation in animal models [13, 61, 62, 63, 64]. Gundogdu et al. investigated the effectiveness of a unique acellular tubular biphasic silk fibroin (BLSF) graft, which comprised a solvent‐cast/salt‐leached sponge combined with a SF film cast, as an alternative urinary conduit in a porcine model of urinary diversion over a 3‐month period. The study results showed that BLSF grafts could facilitate de novo tissue formation and urinary diversion, while improvement in their radial mechanical integrity to maintain urostomy caliber requires further study [63].

Some studies have attempted to improve these properties by blending silk with polyethylene oxide (PEO) to enhance elasticity and the degradation rate without compromising biocompatibility. The addition of PEO has been shown to improve surface morphology, creating porous structures conducive to cell growth and adhesion [41, 43].

### 5.2 Amniotic Membrane in Tissue Engineering

Amniotic membrane has been extensively used in soft tissue engineering due to its antibacterial properties, low antigenicity, and ability to promote cell proliferation and adhesion [19, 24, 65]. Typically, AM is decellularized to eliminate immunogenicity [66]. In our study, the DHAM served as a substrate to decrease inflammation and inhibit stone formation, complementing the silk layers.

### 5.3 Triple‐Layered Scaffold Design

In this study, we investigated a triple‐layered scaffold consists of a SF film, a SF sponge, and DHAM seeded with ADSCs to address the shortcomings of previous scaffolds by offering a multilayered structure that prevents urine leakage, supports cell growth, and reduces inflammation. In the triple‐layered scaffold silk film acts as a barrier layer to prevent leakage of urine, while a porous layer aids in creating the substance for protection of cell growth, and the acellular amniotic membrane is used to decrease inflammation reactions and inhibition of stone formation. Additionally, the collagen hydrogel seeded with ADSCs promotes tissue repair and cell differentiation.

### 5.4 Crystallinity of Silk Scaffolds

Methanol treatment of silk scaffolds can induce proper β‐sheet and α‐helix conformations in SF, thereby enhancing its insolubility and controlling its degradation rate. Silk fibroin primarily exists in crystalline or amorphous (random coil) forms depending on the conditions. Typically, XRD peaks are observed at 12.2°, 19.7°, 24.7°, 28.2°, 32.3° (weak), 36.8° (medium‐weak), and 40.1° (medium‐weak) for the silk I structure, while silk II exhibits peaks at 9.1°, 18.9°, and 20.7° [12, 57].

The prepared silk scaffolds exhibited peaks corresponding to both silk I and silk II structures. Specifically, SF‐S 4% scaffolds displayed peaks at 10.94° and 20.05° associated with silk II, and peaks at 11.91°, 20.37°, and 24.69° corresponding to silk I. Similarly, the SF/0.16% PEO film showed peaks at 12.57° and 19.91°, indicative of silk I and silk II, respectively.

### 5.5 Optimization of Silk Scaffolds With PEO

Jin et al. investigated the effects of polyethylene oxide (PEO) on SF film properties for potential biomedical applications. Their study revealed that the surfaces of all SF/PEO blends exhibited rough and reticulated morphologies due to microphase separation [41]. In particular, the SF/PEO (98/02 wt%) blend showed an evenly dispersed micro‐sized PEO phase. In contrast, films with higher PEO content (10–40 wt%) displayed a sparser morphology, as observed in cross‐sectional analyses [41].

Brian D. Lawrence et al. evaluated the structural and morphological features of SF films with varying concentrations of PEO [43]. At 0.05% wt vol^−1^ PEO, the film surfaces exhibited porous void features, while all other PEO concentrations resulted in globule‐like surface features. These findings suggest that PEO molecules were not uniformly dispersed in the aqueous phase at concentrations below 0.20% during film casting. At 0.20% PEO and above, the surfaces of silk films were entirely composed of uniformly sized fibroin globules. Notably, the SF/0.16% PEO film demonstrated a pore‐like morphology [43].

In general, polyethylene oxide is blended with SF to improve the mechanical properties and biocompatibility of the silk films. Additionally, the presence of PEO in the silk film affected the morphology, which corresponded to the concentration of PEO. Such surface patterns can be used as suitable substrates for cell growth and may promote cell adhesion.

### 5.6 Mechanical Properties

Previous studies have shown that blended silk/PEO films can be prepared with suitable UTS, EM, and elongation, making them effective for protecting bladder tissue [41, 43]. Despite the high EM and UTS of the films, they not only presented no disadvantages but also demonstrated a superior role in bladder augmentation. Compared to the human bladder, the sponges exhibited similar UTS and ultimate strain but had a significantly higher EM (3.6‐fold greater) [15, 16].

Research has also demonstrated that DHAM possesses high EM and UTS but limited elongation [50]. The elongation of films and sponges was comparable, which is attributed to the reduced film thickness and low sponge concentration, making them suitable for bladder applications.

Tu et al. studied acellular scaffolds made from Bombyx mori SF, creating two bi‐layer configurations: one with solvent‐casting/salt leaching alone (Group 1) and the other combining this with silk film casting (Group 2) [15]. Both groups supported tissue regeneration in a porcine model, showing UTS similar to controls but with increased stiffness (higher EM). De novo tissue formation was noted, with tissue characteristics at 80% and 46% of control levels for Groups 1 and 2, respectively [15].

Seth et al. evaluated silk biomaterials using gel spinning and a combined method in a rat model. The combined method (FF group) had lower UTS and EM compared to gel‐spun matrices but was four times more elastic, which may benefit the treatment of distensible bladder defects [16].

Overall, mechanical testing showed that the scaffolds' UTS and elongation were comparable to human bladder tissue, though they had a higher EM. Previous studies suggest that a higher EM may not adversely affect bladder function and could improve outcomes in animal models [59, 60].

The triple‐layer scaffold, composed of film, sponge, and DHAM, was designed to mimic bladder tissue. Its UTS, ultimate strain, and elongation matched those of the human bladder, although its EM was significantly greater (7.5‐fold higher), largely due to the presence of the DHAM and film layers. Notably, previous studies have shown that a high EM does not have adverse effects and has yielded promising results in both small and large animal models.

The elongation of the triple‐layer scaffold was greater than that of the individual components (SF film, SF sponge, and DHAM). This increased elongation is due to the need for an initial force to stretch the three layers together. Once the initial force was applied, the scaffold resisted stretching until the layers began to rupture. This layered design contributed to the scaffold's superior mechanical behavior and its potential for bladder tissue engineering.

Generally, various biomechanical tests have been employed to study the behavior of bladder tissue. Methods such as uniaxial tensile tests, biaxial tensile tests, and dynamic mechanical analysis are among the techniques used to assess the mechanical properties of tissues. Despite the availability of multiple testing techniques, the uniaxial test remains one of the most commonly used methods. This is because of its cost‐effectiveness, simplicity, and efficiency. The uniaxial test allows for precise and controlled testing in specific directions and regions, providing valuable information about tissue behavior [7, 60]. Our focus in this study was to investigate the mechanical properties of our scaffold under static conditions, utilizing the basic uniaxial tensile test. This test involves applying mechanical stress to a strip of the scaffold in one direction. While the biomechanical behavior of the bladder is characterized by its ability to store urine at low pressure and then contract to expel it, it should also be noted that the bladder must withstand dynamic forces at body temperature.

In this study, we utilized the mechanical properties of human and rat bladders from previous reports, although we attempted to recreate the same experimental conditions [50, 59, 60]. Nevertheless, there may be differences in experimental conditions and tissue preparation methods, such as the size of the samples, sample shape, test temperature, and other factors. These factors could have an impact on the applicability of the study's results, which must be considered.

### 5.7 Cell Viability of the Scaffolds

According to H&E staining, the protocol of decellularization of the HAM eliminated cells from membrane absolutely and prepared DHAM as a substrate for cells, similar to the previous study [50].

Moreover, cell viability on SF‐S 4% with collagen was considerably higher in comparison to SF‐S 4% and 6%, which showed suitable cell preservation associated with collagen hydrogel. In summary, the percentage and size of porosity of SF‐S 4% were better than those of SF‐S 6% for cell survival. In addition, DHAM as a native tissue made a good substrate, and blend film was shown to be suitable for proliferation of ADSCs.

In addition, FESEM images showed that ADSCs were attached to the surfaces of all scaffolds after 72 h of cell culture. ADSCs rapidly spread, branched, and elongated into sponges, especially the SF‐S 4%, with collagen hydrogel.

### 5.8 pH and Stone Formation

It is known that the pH of human urine ranges between 4.5 and 7.8, with an average of around 6. At higher pH levels, the risk of forming apatite stones increases, while lower pH may promote uric acid stone formation. Optimizing the scaffold's interaction with urine is crucial to prevent stone formation [67].

Scaffolds were exposed to urine in two groups to evaluate stone formation after implantation of scaffolds in the bladder. In the first group, DHAM exposed urine (three layered scaffold), and in the secondary group, SF sponge was in touched by urine (two layered scaffold). FESEM images show particles in the two‐layer group, while there aren't any particles in the three‐layer group (Figure 10). These separated particles from sponge, when flowed in urine, can form crystals and stones. Therefore, when a DHAM layer contacts to urine, it acts as a barrier to prevent direct contact with silk sponge and stone formation. Direct contact of sponge to urine led to flowing silk particles in urine and increased the risk of stone and crystalline formation.[15, 16, 17] Furthermore, during the testing period, there was no decrease in urine volume observed, indicating that the scaffold groups did not have leakage due to the presence of the SF film layer, which functions as a barrier in the triple‐layered scaffold.

### 5.9 Comparison with Previous Studies

Previous studies using bi‐layer silk scaffolds in rat and pig models have reported issues such as scaffold degradation, unoptimized mechanical properties, stiffness, and inflammatory reactions [15, 16, 17, 63, 64]. To improve upon these designs, we reduced the silk sponge concentration (to 4%) to increase porosity and enhance cell survival while adjusting the film thickness (∼140 µm) and incorporating PEO to improve mechanical properties and biodegradation rates. Additionally, we used DHAM to minimize inflammatory reactions, and ADSCs were seeded with collagen hydrogel to support cell growth and tissue regeneration.

In our study, we developed a triple‐layered composite scaffold containing a film made of SF/PEO, a sponge of SF, and a DHAM. The obtained uniaxial tensile test data, along with FTIR and XRD analysis on the scaffolds, indicated that the inclusion of PEO enhanced the flexibility and biodegradability of the film made of SF. This result was achieved owing to the increased hydrophilicity of this polymer, which improved the film's compatibility with biological systems, while maintaining the mechanical properties of the scaffold, thus providing it with sufficient stiffness, tensile strength, and elongation. Overall, these characteristics made it an ideal candidate for use as a scaffold in tissue engineering applications, particularly for tissue regeneration in organs with biomechanical loads.

FESEM images and MTT assay confirmed that the addition of PEO in the composition of the SF films improved the morphology of the film, which led to increased cell survival. The resulting films displayed a globule‐like morphology, which was found to be beneficial for cell survival as it provided a more suitable environment for cell attachment and growth. Overall, the inclusion of PEO in the production of the SF/PEO films improved the film's morphology, which contributed to enhanced cell survival.

To further enhance the compatibility of the scaffold with living cells, we incorporated a sponge layer into the structure, which provided a favorable environment for the cultivation of cells. A collagen hydrogel was utilized to encapsulate ADSCs, which were then cultured on the sponge layer. Based on DAPI staining and MTT assay results, this created a conducive environment for supporting cell survival and the preservation of their viability.

Furthermore, we incorporated a DHAM layer in the inner layer of the scaffold to provide a barrier between the SF scaffold layers and urine. DHAM has demonstrated exceptional biocompatibility and cell‐friendly properties, making it an ideal material for use in this context. Additionally, based on the pH measurement of urine and FESEM imaging after the scaffold groups were placed in the stone formation chamber, using DHAM may help prevent potential issues such as stone formation and inflammatory reactions, as the scaffold's inner layer made of DHAM would act as a barrier to urine exposure. Additionally, this setup helps protect the underlying cells from any potential negative effects that could be caused by direct exposure to uric substances.

In summary, the scaffold was designed in three layers. First, the SF/0.16% PEO film was utilized to create a leak‐preventive and optimized layer, ensuring improved mechanical properties and degradation rate. Second, the SF/S 4% sponge layer was incorporated as a cell‐supporting layer, encapsulated with ADSCs in a collagen hydrogel to facilitate and maintain cell survival. Finally, the inner layer was constructed using DHAM, which offered an outstanding biocompatible and non‐inflammatory surface, reducing the risk of stone formation due to silk layers breaking down within the urine. Unwanted fragments of silk layers, resulting from the degradation process, can indeed lead to stone formation when exposed to urinary substances. By incorporating DHAM in the inner layer, we effectively protect the scaffold from direct contact with urine, thereby preventing stone formation within the bladder.

The aim of the present study was to fabricate a triple‐layer scaffold for bladder tissue engineering. Also, we can co‐culture urothelial cells with ADSCs to aid cell differentiation and tissue repair. We should follow up on animal models for a long time and investigate the issue of stone formation in the bladder.

### 5.10 Limitations and Opportunities for Improvement

While this study contributes valuable insights to the field, it is important to acknowledge its limitations and identify areas for potential improvement. First, the mechanical properties of human and rat bladders were used as references based on previous reports [50, 59, 60]. However, it is important to note that while we attempted to recreate the same experimental conditions, there is likely to be some variation in factors such as the size of samples, sample shape, and tissue temperature. Additionally, from a mechanical biology perspective, the bladder is strongly influenced by the mechanical environment as mechanical factors have an impact on the entire body and organs' biological processes, including positive functional adaptation reconstruction, negative structural damage, and disease occurrence. This study investigated bladder mechanical properties under uniaxial and static condition, which represents a significant limitation, as the mechanical properties of the bladder in a dynamic situation, similar to that found in the body, should be assessed before a preclinical study is conducted.

Second, the present study did not evaluate the degradation behavior of the scaffolds in detail. While we assessed the degradation tendencies based on the two primary silk structures (silk I and silk II) after methanol treatment and the addition of PEO to the SF film layer, leading to an optimized structure for a desirable degradation rate a comprehensive investigation of the degradation profile during the degradation process, either in vitro or in vivo, could provide essential data to support the practical applicability of the scaffold.

Additionally, confirming the identity of MSCs requires the use of specific cellular markers according to the Mesenchymal and Tissue Stem Cell Committee of the International Society for Cellular Therapy (ISCT). In the present study, we followed a previously published protocol for the characterization of ADSCs [51]. According to the referenced study, the necessity for ADSC characterization was addressed using cells isolated from the subcutaneous and inguinal adipose tissues of adult Wistar rats. Based on prior reports, a positive marker expression above 85% and a negative expression below 5% are indicative of MSCs, distinguishing them from hematopoietic cells [51]. In our study, flow cytometry analysis showed that the isolated cells were positive for the MSC markers CD90 (86.3%) and CD105 (98.5%), and negative for the hematopoietic marker CD34 (2.27%). These results confirm that the isolated cells were MSCs. Although our immunophenotyping of ADSCs was limited in comparison to the full criteria recommended by the ISCT, we adhered to a validated and published protocol for ADSC isolation and culture.

Lastly, cell survival, adhesion, and morphology were evaluated using MTT assay, DAPI staining, and FESEM. Based on these assessments, we observed high levels of cell viability and adhesion on both DHAM and SF/S 4% combined with collagen hydrogel. However, it is important to note that while these tests provide preliminary insights into cell behavior, they are not sufficient to fully confirm biocompatibility. Additional assays, such as F‐actin staining for cytoskeletal organization and live/dead staining for viability would strengthen our findings. Therefore, further evaluations are necessary to provide conclusive evidence supporting the scaffolds biocompatibility.

## 6 Conclusion

The aim of this study was to fabricate a triple‐layered scaffold for bladder tissue engineering by incorporating a bilayer SF scaffold, DHAM, collagen hydrogel, and ADSCs. The findings from this current report indicate that a triple‐layered scaffold can support the formation of neotissue in a diseased bladder model and would be expected to have minimal inflammatory reactions and appropriate degradation timelines. The bladder is a complex organ consisting of multiple layers, making a triple‐layered scaffold potentially more accurate and acceptable for bladder regeneration. In summary, the structural, mechanical, and biological attributes of this scaffold are deemed promising and could offer a functional substance for bladder tissue engineering applications.

We would like to emphasize that as part of our in vitro research, we have performed thorough testing on the produced scaffold. In the future, we plan to broaden our research by carrying out tests on animal models, which will give important insight into the performance and efficacy of the scaffold in a living organism. This extension of our research will enable us to evaluate the practicality and efficiency of the scaffold in a more realistic environment. In summary, at the next stage, we aim to perform bladder augmentation on rat models using the optimized triple‐layered scaffold made of SF/0.16% PEO, SF‐S 4%, and DHAM for a period of at least 3 months. We will carry out histological, immunohistochemical, urodynamic evaluations, and mechanical tests to provide critical information for future studies that could be conducted using larger animal models.

## Author Contributions

Conceptualization: Z. Hassannejad and M. Shafieian; Funding acquisition and resources: M. Shafieian and Z. Hassannejad; Experimental testing: M. Mamdoohi; Data analysis: M. Mamdoohi and Z. Hassannejad; Manuscript preparation: M. Mamdoohi; Manuscript editing: M. Shafieian and Z. Hassannejad. All authors have read and agreed to the published version of the manuscript.

## Conflicts of Interest

The authors declare no conflicts of interest.

## Supporting information




**Supporting file**: mabi70059‐sup‐0001‐DataFile.xlsx

## Data Availability

The data that support the findings of this study are available from the corresponding author upon reasonable request.
